# LncRNA OSER1-AS1 regulates the inflammation and apoptosis of rheumatoid arthritis fibroblast like synoviocytes via regulating miR-1298-5p/E2F1 axis

**DOI:** 10.1080/21655979.2022.2037854

**Published:** 2022-02-14

**Authors:** Qiang Fu, Mei-Jie Song, Jing Fang

**Affiliations:** a Department of Rheumatology and Immunology, Jiangxi Provincial People’s Hospital Affiliated to Nanchang University, Nanchang, Jiangxi, China; b Geriatric Department, Jiangxi Provincial People’s Hospital Affiliated to Nanchang University, Nanchang, Jiangxi, China

**Keywords:** OSER1-AS1, miR-1298-5p, E2F1, rheumatoid arthritis

## Abstract

It has been reported that long noncoding RNAs (LncRNAs) take part in the progression and occurrence of rheumatoid arthritis (RA). The current work aimed to dig the effect of lncRNA OSER1-AS1 on RA and the associated mechanism. Quantitative real-time polymerase chain reaction (qRT-PCR) was made to decide that OSER1-AS1 was significantly lowly expressed in synovial tissue and serum of RA patients, which was consistent in RA-FLSs cell lines. The result of ROC curve indicated that OSER1-AS1 could be a diagnostic biomarker for RA patients. Cell Counting Kit-8 assay (CCK-8), EdU staining and flow cytometry were performed to explore the effect of OSER1-AS1 on RA-FLSs in vitro. Relative levels of interleukin-1 (IL-1), interleukin-6 (IL-6), matrix metalloproteinases-3 (MMP-3) were detected by ELISA and the result displayed that overexpression of OSER1-AS1 inhibited RA-induced inflammatory production of IL-1, IL-6 and MMP3. Bioinformatics analysis, luciferase reporter, RNA immunoprecipitation assays (RIP) and RNA pull-down assay were conducted to confirm the binding between microRNA-1298-5p (miR-1298-5p) and OSER1-AS1 or E2F transcription factor 1 (E2F1). Mechanistically, OSER1-AS1 serves as a competing endogenous (ceRNA) in RA-FLSs through the sponge of miR-1298-5p and increase in the expression of E2F1. Further restoration experiments revealed that miR-1298-5p mimics and E2F1 silencing could partially reverse the inhibiting effect of OSER1-AS1 overexpression on propagation and apoptosis in RA-FLSs. The results illustrated the biological mechanism of OSER1-AS1/miR-1298-59/E2F1 axis in RA progression. The outcomes indicated that OSER1-AS1 might be adopted as a hopeful diagnostic and therapeutic objective for RA.

## Graphical abstract

**Figure:**
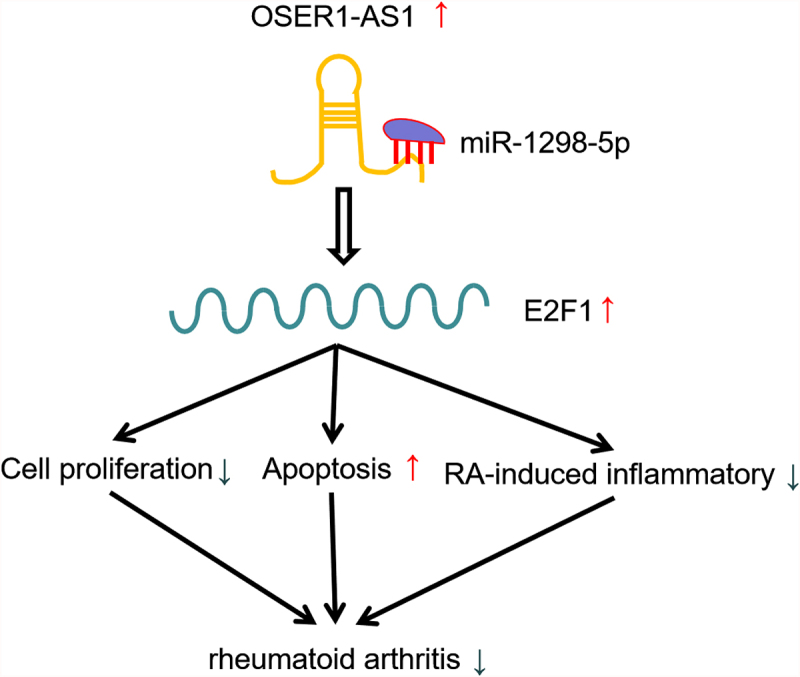

## Introduction

Rheumatoid arthritis (RA), a frequent chronic inflammatory autoimmune disease, has the characteristics such as progressive joint inflammation and articular cartilage destruction, which finally leading to serious joint deformity and disability [1–3]. Regardless of great advancement in the diagnosis and treatment for RA, increasing studies indicate that the prevalence of RA continues to increase in recent these years, which largely impair quality of life of RA patients and cause serious social and medical burden. Numerous studies were performed to explore the pathological mechanisms of RA progression, the pathogenesis of RA was still mysterious. Henc, it is vital to further illustrate the core molecular system of RA, which may contribute to providing novel diagnostic and therapeutic objectives for treating RA.

Long non-coding RNAs (lncRNAs) are a team of non-coding RNA with length over 200 nucleotides. According to increasing evidence, several lncRNAs were differentially expressed in RA and participated in the onset and development of RA. Piao and coworkers found that 190 lncRNAs were upregulated and 131 lncRNAs were downregulated in RA synovial tissues [4]. Subsequently, they identified that lncRNA RP11-83J16.1 promoted cell propagation, move, aggression and inflammation in RA fibroblast-like synoviocytes (RA-FLS) [5]. Zou *et al*. found that lncRNA LERFS was downregulated in RA-FLS and it may be adopted as a promising therapeutic objective for patients suffering from RA [6]. Peng and colleagues demonstrated that lncRNA GAS5 inhibited RA inflammation in synovial tissue by controlling HDAC4 by miR-128-3p, which suggested that GAS5/miR-128-3p/HDAC4 axis had therapeutic potential in RA treatment [7]. As a newly identified lncRNA, OSER1-AS1 was verified to be related to the pathogenesis of some malignant tumors, including heap to cellular carcinoma [8] and non-small cell lung cancer [9–11]. Interestingly, a current research found that OSER1-AS1 was greatly downregulated in RA [5], but the hidden effects and mechanism of OSER1-AS1 on RA were unknown greatly.

miRNAs were a class of non-coding RNAs, which regulated biological functions by inhibiting the expression of targeted mRNAs. Several studies indicated miRNAs took part in the pathogenesis of RA. Wang *et al*. found the downregulation of miR-431-5p in RA synovial tissues and FLSs and miR-431-5p regulated cell propagation, apoptosis, and cell cycle of RA FLSs via targeting XIAP [12]. Jiang *et al*. displayed that miR-147b-3p promoted synovial inflammation through repressing ZNF148 in RA [13]. Previous studies displayed that miR-1298-5p participated in the pathogenesis of some cancers such as non-small cell lung cancer [14], breast cancer [15], and bladder cancer [16]. Nevertheless, the functions and mechanisms of miR-1298-5p remained to be further elucidated. E2F1 was verified to be downregulated in RA and overexpression of E2F1 inhibited p53 signaling pathway to suppress the propagation and aggression of FLS and the generation of pro-inflammatory cytokines in RA [17]. However, there were no available studies exploring the association between miR-1298-5p and in RA.

The recent research identified that OSER1-AS1 was greatly lowly-expressed in RA. Overexpression of OSER1-AS1 inhibited FLSs propagation, inflammation and boosted apoptosis in RA. OSER1-AS1 acted as the sponge of miR-1298-5p and E2F1 was the downstream targeted mRNA of miR-1298-5p. Thus, we put forward the hypothesis that OSER1-AS1 might be involved in the biological processes in RA-FLSs through miR-1298-5p/E2F1 pathway. The present work aimed to be further confrmed pathogenesis of RA and find a potential target for RA patients in the future.

## Materials and methods

### Clinical samples

30 tissue and blood samples from RA patients (female 20, male 10, average age 45.5 ± 5.4 year) and compared 30 traumatic patients conducted with amputations (female 13, male 17, average age 40.2 ± 6.7 year) at Jiangxi provincial people’s hospital Affiliated to Nanchang University from June 2016 to January 2021. Synovia tissue samples were frozen instantly and conserved in liquid nitrogen after collected during surgery, and blood sample were processed in 6 hours. All patients with RA were diagnosed by the 2010 American College of Rheumatology (ACR)/European League Against Rheumatism (EULAR) [18]. Patients suffering from severe systemic diseases were excluded. The research was agreed by the ethical commission of Jiangxi people’s hospital affiliated to Nanchang University (Approval number: 2021–70) and all patients enrolled had offered the consent form before the research.

### Cell culture

RA-FLSs and 293 T cells lines were bought from Shanghai Institute of Biochemistry and Cell biology, followed by culture in Dulbecco altered Eagle medium (DMEM) (Gibco, Grand Island, NY) added with fetal bovine serum (FBS) of 10% (HyClone, Logan, UT), and 1% antibiotics (100 U/ml penicillin and 100ug/ml streptomycin). Both cell lines were maintained in the 5% CO2 and 37°C incubator.

### Cell transfection

The full length sequences of OSER1-AS1 were combined into pcDNA3.1 vector (Invitrigen, Carlsbad, Calif, USA) to produce pcDNA-OSER1-AS1. Two separate siRNAs targeting OSER1‐AS1 (si1- OSER1‐AS1 and si2- OSER1‐AS1), si-NC, shRNAs against E2F1, non-targeting shRNA (sh-NC), miR-1298-5p simulates and negative control (miNC) matched were provided from Guangzhou Ruibo Biotechnology (Guangzhou, China). The orders of sh-E2F1 were listed in ***Supplementary Table S2***. RA-FLSs and 293 T cells lines were transfected with pcDNA3.1 Vector, pcDNA-OSER1-AS1, NC, miR-1298-5p mimics and sh-E2F1 by using Lipofectamine 2000^TM^ reagent (Thermo Fisher Scientific) on basis of the manufacturer’s guidance.

### Cell counting kit-8 (CCK-8) assay

Cells were harvested at 48 hours after transfection and seeded into 96-wells plates (1.5 × 10^3^ cells per well). Cells were culture for five time points: 0, 24, 48, 72 and 96 h. Cell proliferation was assessed through the incubation of cells with 10ul CCK-8 reagent (Doindo, Japan) for 2 h. Then, the optical density was measured at the wavelength of 450 nm by using a microplate reader (Thermo Fisher Scientific, Inc.).

### EdU staining

The seeding of RA-FLSs into 96-well plates was made at a density of 5 × 10^3^ cells/well, followed by culture with a 100ul culture medium containing 50umol/l EdU for 2 h. Afterward, fixed by 4% Paraformaldehyde Fix Solution (PFS), cells were mixed with 50ul glycine (2 mg/ml) for 5 min and 100ul 0.5% TritonX-100 for 10 min incubation. Subsequently, away from light, the incubation of cells was made with 100ul Apollo reaction staining solution at room temperature for 30 min. Discarded Apollo staining solution and added 100ul 0.5% TritonX-100 for 10 min incubation (repeated for 2–3 times). Next, away from light, 100ul Hoechst33342 reaction solution was added for incubation at room temperature for 30 min. At last, the quantity of proliferated cells (EdU-positive cells) and total cells (Hoechst33342-positive cells) was calculated by capturing four random fields with a fluorescence microscope (Olympus)

### Real-time quantitative reverse transcription polymerase chain reaction (RT-qPCR)

miRNeasy Serum/Plasma Kit (QIEGEN, Germany) was adopted to extract total RNAs from blood samples, and Trizol reagent (Invitrogen, USA) was utilized to extract tissue sample and cells. The Prime Script™ RT reagent kit was employed to perform reverse transcription reactions of OSER1-AS1 and E2F1 on basis of the manufacturer’s guidance (Invitrogen, USA). The reverse transcription reactions of OSER1-AS1 and E2F1 mRNA were carried out riboSCRIPT Reverse Transcription Kit (RiboBio, China). While, the reverse transcription reactions of miR-1298-5p were conducted with riboSCRIPT Reverse Transcription Kit (RiboBio, China). RT-qPCR analyzes were carried out using SYBR Premix Ex Taq kit (Takara, Japan). Glyceraldehyde-3-phosphate dehydrogenase (GAPDH) and U6 were applied as the internal control for reference genes. The 2^−ΔΔCt^ approach [19] was employed for the calculation of relative expression levels of miRNA and mRNA. ***Supplementary Table S1*** lists the sequences of the primers.

### Western blot assay

RIPA lysis buffer (Beyotime Biotechnology, Shanghai, China) wa employed to lyse tissue samples and cells, and BCA Protein Assay Kit (Beyotime, Jiangsu, China) was adopted to quntify the overall protein concentration. Protein was isolated applying SDS-PAGE of 10% and then electro-transferred to PVDF membranes. Next, the one-hour blocking of PVDF membranes was made with fat-free milk at room temperature, the overnight incubation of PVDF membranes was conducted with primary antibodies at 4°C, and the one-hour incubation of secondary antibodies was conducted at room temperature. Cell Signaling Technology (Danvers, MA, USA) offered E2F1 and GAPDH primary antibodies. The visualization of immunoreactive bands was made with improved chemiluminescence kit (Thermo Fisher Scientific) on basis of the guidance of the manufacturer, and ImageJ software (NIH, USA) was adopted to quantify the intensity of protein bands.

### Flow cytometry analysis

Annexin V-APC/PI Apoptosis Detection Kit (KeyGEN, China) was employed to make flow cytometry analysis for apoptosis. Cells were harvested at 48 hours after transfection, followed by resuspending in 500ul binding buffer. Then, away from light, 5ul Annexin V-APC and 5ul propidium iodide (PI) were put at room temperature for 15 min. Cell apoptosis was determined using CytoFLEX Flow Cytometer (Beckman, USA) within a 1 h period and data was analyzed using FlowJo software.

### ELISA

The collection of cell supernatant of RA-FLSs was made, and ELISA kits (Thermo Fisher Scientific) were employed to detect the relative levels of interleukin-1 (IL-1), interleukin-6 (IL-1) and matrix metalloproteinases-3 (MMP-3). Human ELISA kits for IL-1 (Invitrogen), IL-6 (Invitrogen) and MMP3 (Invitrogen) were applied on basis of the manufacturer’s instructions.

### Dual luciferase reporter gene assay

The target miRNAs of OSER1-AS1 were analyzed by querying Starbase (http://starbase.sysu.edu.cn/) [20], which displayed that there was a putative target for miR-1298-5p in OSER1-AS1. The synthesis and clone of wild kind (OSER1-AS1-wt) and mutant OSER1-AS1 (OSER1-AS1-mut) binding sequences for miR-1298-5p into pGL3 reporter vector (Promega, Madison, WI, USA) were made. In the meantime, TargetScan indicated that E2F1 was a downstream objective for miR-1298-5p [21]. Besides, to evaluate the relationship between miR-1298-5p and OSER1-AS1 or E2F1, the co-transaction of 293 T and RA-FLSs cells was made with pGL3 reporter luciferase vector, which containing the 3’-UTR sequence of E2F1-mut and E2F1-wt or RA-FLSs -wt and RA-FLSs -mut and miR-1298-5p mimics or negative control applying Lipofectamine 2000^TM^ reagent (Thermo Fisher Scientific). Luciferase Reporter Kit (Promega) was adopted to measure relative luciferase activities on basis of the instructions of manufacture.

### RNA pull-down

Biotin-labeled WT-miR-1298-5p, Mut-miR-1298-5p and negative management were purchased from GenePharma (China). The transfection of RA-FLSs was made with biotin-labeled WT-miR-1298-5p, Mut-miR-1298-5p and negative management and harvested at 48 h after transfection. The lysis and incubation of cells were made with the streptavidin magnetic beads for 30 min. The enrichment of RNA was examined with RT-qPCR.

### RNA immunoprecipitation assays (RIP) assay

The RIP assay was performed to detect the binding of miR-1298-5p and OSER1-AS1 or E2F1 using RIP kit (Millipore, MA). After lysis with Pierce IP Lysis Buffer (Thermo Scientific), the incubation of RA-FLSs cells was made with anti-AGO2 (Millipore) and anti-IgG (Millipore) antibodies. TRIzol reagent was used to extract RNA in the sample and input for subsequent qRT-PCR analysis to evaluate the expression extent of OSER1-AS1.

## Statistical analysis

**Receiver running feature curve exploration** A ROC curve was generated to assess the precision of OSER1-AS1 expression to distinguish the RA patients from healthy subjects. ROC curve analysis was conducted by using GraphPad Prism 8.

Data was shown as mean ± standard deviation (SD) and representative of 3 independent tests. The comparison of diversities between two groups with student’s t test was made. One-way analysis of variance (ANOVA) was adopted to explore the statistical significant differences among multiple groups. Statistical analysis adopted SPSS 22.0 software (SPSS, Chicago, IL, USA). P < 0.05 was of statistical significance.

## Results

### OSER1-AS1 was lowly expressed in RA patients and cell lines

**Figure 1.**
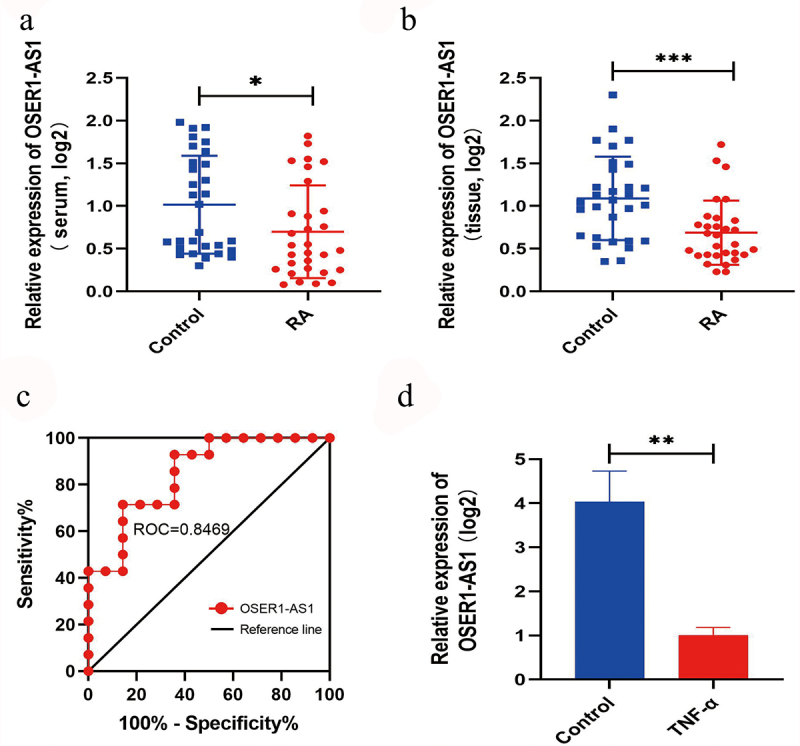
OSER1-AS1 was lowly expressed in RA samples. (a-b) OSER1-AS1 was lower in serum samples and synovial tissues of RA patients(n = 30) and the compared healthy controls(n = 30). (c) ROC analysis indicated that the expression level of OSER1-AS1 in serum samples could be a diagnostic biomarker for RA. (d) Expression levels of OSER1-AS1 in TNF-α-induced RA-FLSs. All experiments were conducted in triplicate. *p < 0.05, **p < 0.01, ***p < 0.001.

The relative extents of OSER1-AS1 in serum and synovial tissues of RA patients and compared healthy management were investigated by RT-qPCR. OSER1-AS1 was lower in serum samples and synovial tissues of RA patients (n = 30) (Figure 1a) and the compared healthy controls (n = 30) (Figure 1b). Besides, a receiver running feature curve was drawn to assess the precision of gene expression to tell apart RA patients from healthy controls (Figure 1c). Furthermore, OSER1-AS1 was significantly lowly expressed in TNF-α-caused RA-FLSs (Figure 1d).

### Overexpression of OSER1-AS1 inhibited the propagation, release of inflammatory factor and drove the apoptosis of TNF-α-induced RA-FLSs

**Figure 2.**
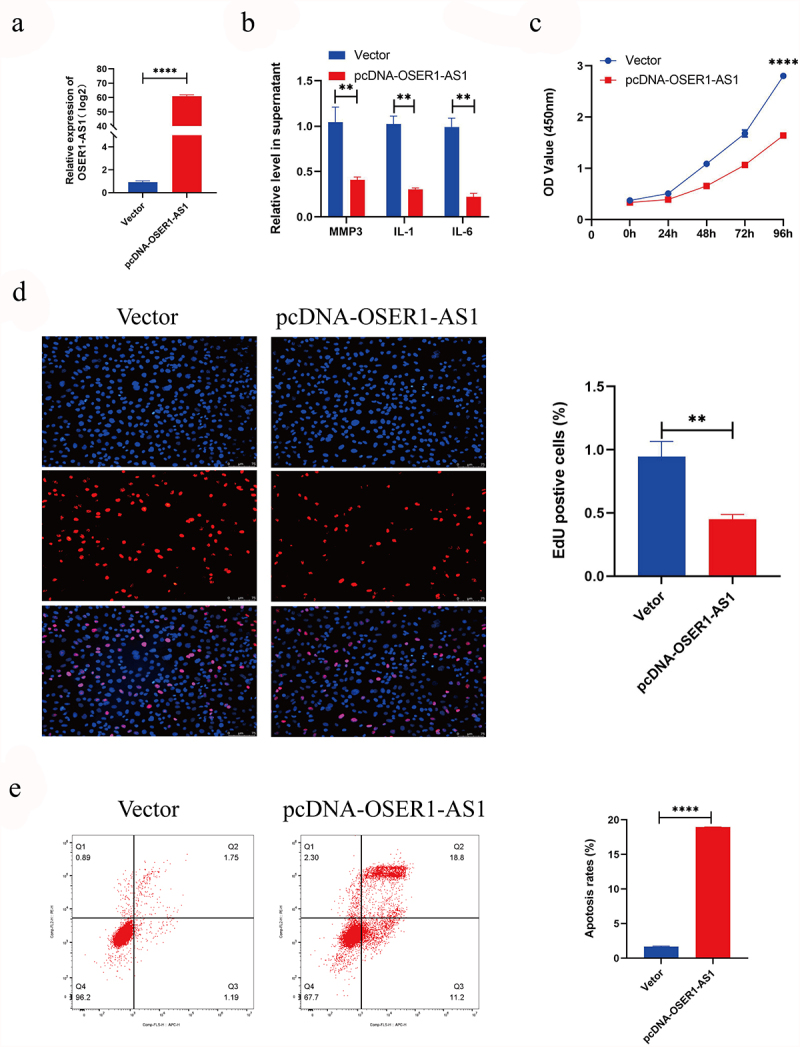
Overexpression of OSER1-AS1 inhibited the proliferation, release of inflammatory factor and promoted the apoptosis of TNF-α-induced RA-FLSs. (a) qRT-PCR analysis was performed to assess the overexpression efficiency of pcDNA-OSER1-AS1 after transfected with pcDNA-OSER1-AS1 and Vector. (b) Relative levels of interleukin-1 (IL-1), interleukin-6 (IL-6), matrix metalloproteinases-3 (MMP-3) were detected by ELISA. (c-d) The proliferation of the TNF-α-induced RA-FLSs was measured by CCK8 assay and EdU staining. (e)The cell apoptosis of the TNF-α-induced RA-FLS was detected by Flow cytometry apoptosis assay. All experiments were conducted in triplicate. ***p < 0.001, ****p < 0.0001.

To explore the effect of OSER1-AS1 on RA-FLSs, RA-FLSs were transfected with pcDNA-OSER1-AS1 and vector. And the outcomes of RT-qPCR displayed that OSER1-AS1 expression level was greatly upregulated in RA-FLSs (Figure 2a). The result of ELISA showed that overexpression of OSER1-AS1 inhibited RA-induced inflammatory production of IL-1, IL-6 and MMP3 (Figure 2b). The outcomes of CCK8 assay displayed that the viability of proliferation of RA-FLSs cells was significantly inhibited after transfected with pcDNA-OSER1-AS1 (Figure 2c). Furthermore, the result of the EdU staining showed the same tendency (Figure 2d). In the meantime, Flow cytometry apoptosis assay was employed to examine the cell apoptosis of the TNF-α-caused RA-FLSs, and the result displayed that the percent of apoptosis cells was obviously grown by overexpression of OSER1-AS1 (Figure 2e).

### Knockdown of OSER1-AS1 accelerated the proliferation and suppressed the apotosis of TNF-α-induced RA-FLS

In order to further investigate the effect of OSER1-AS1 on RA-FLS, RA-FLS cells were transfected with si-OSER1-AS1 or si-NC. The results of RT-qPCR showed that the expression of OSER1-AS1 was significantly reduced by si-OSER1-AS1 (Supplementary Figure S1A). ELISA assay showed that silence of OSER1-AS1 stimulated RA-induced inflammatory production of IL-1, IL-6 and MMP3 (Supplementary Figure S1B). The outcomes of CCK8 assay and the EdU staining displayed that the viability of proliferation of RA-FLSs cells was significantly promoted after transfected with si-OSER1-AS1 compared with si-NC (Supplementary Figure S1C-D). In the meantime, the results of Flow cytometry apoptosis assay the cell apoptosis of the TNF-α-caused RA-FLSs was obviously inhibited by silence of OSER1-AS1 (Supplementary Figure S1E).

### OSER1-AS1 predominantly sponges miR-1298-5p

**Figure 3.**
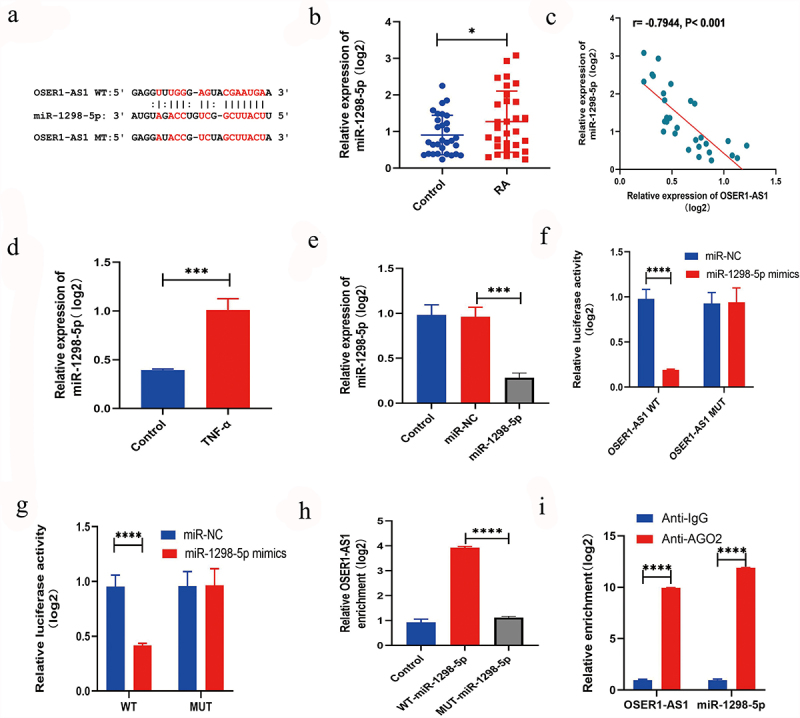
LncRNA OSER1-AS1 directly targets miR-1298-5p in TNF-α-induced RA-FLS. (a) Binding sites between OSER1-AS1 and miR-1298-5p are predicted by StarBase. (b) miR-1298-5p was overexpressed in synovial tissues of RA patients compared with healthy controls. (c) The expression levels of OSER1-AS1 and miR-1298-5p in synovial tissues of RA patients were negatively correlated. (d) miR-1298-5p expression was signifcantly increased in TNF-α-induced RA-FLSs. (e) miR-1298-5p could be upregulated by the miR-1298-5p mimics. (f) Dual-luciferase reporter assay was applied to confirm the binding between OSER1-AS1 and miR-1298-5p in 293 T cells and co-transfection with OSER1-AS1-wt and miR-1298-5p mimic greatly reduced the luciferase activities compared with OSER1-AS1-mut and miR-1298-5p mimic. (g) Dual-luciferase reporter assay was applied to confirm the binding between OSER1-AS1 and miR-1298-5p in RA-FLSs. (h) The interaction between OSER1-AS1 and miR-1298-5p detected by RNA pulldown assay and the expressions of miR-1298-5p in biotinylated OSER1-AS1 was enriched. (i) The expression of miR-1298-5p and OSER1-AS1 was determined by RIP assay. Each experiment was performed in triplicate. The data were shown as mean ± SD. N = 3, *p < 0.05, ***p < 0.001, ****p < 0.0001.

To further examine the system of OSER1-AS1 in RA-FLSs, the target miRNAs of OSER1-AS1 were predicted by querying Starbase (http://starbase.sysu.edu.cn/) [16], which showed that there was a putative objective for miR-1298-5p (Figure 3a) in OSER1-AS1. Furthermore, the expression extent of miR-1298-5p was greatly higher in synovial tissues of RA patients by comparing with healthy controls (Figure 3b). A relationship analysis displayed a negative relationship between the expression extents of OSER1-AS1 and miR-1298-5p in synovial tissues of RA patients (Figure 3c). In addition, miR-1298-5p expression was greatly increased in TNF-α-induced RA-FLSs (Figure 3d). Otherwise, the expression of miR-1298-5p could be increased by the miR-1298-5p mimics (Figure 3e). Dual-luciferase reporter assay was applied to confirm the binding between OSER1-AS1 and miR-1298-5p in 293 T cells and co-transfection with OSER1-AS1-wt and miR-1298-5p mimic singificantly decreased the luciferase activities by comparing with OSER1-AS1-mut and miR-1298-5p mimic (Figure 3f). Meanwhile, dual-luciferase reporter assay was applied to confirm the binding between OSER1-AS1 and miR-1298-5p in RA-FLSs (Figure 3g). The interaction between OSER1-AS1 and miR-1298-5p detected by RNA pull-down assay and the outcome displayed that the expressions of miR-1298-5p in biotinylated OSER1-AS1 were enriched (Figure 3h). Moreover, the expression of miR-1298-5p and OSER1-AS1 was decided by RIP assay. All the results suggested that OSER1-AS1 can sponge miR-1298-5p in RA-FLSs.

### miR-1298-5p targeted E2F1 and had a negative correction with E2F1

**Figure 4.**
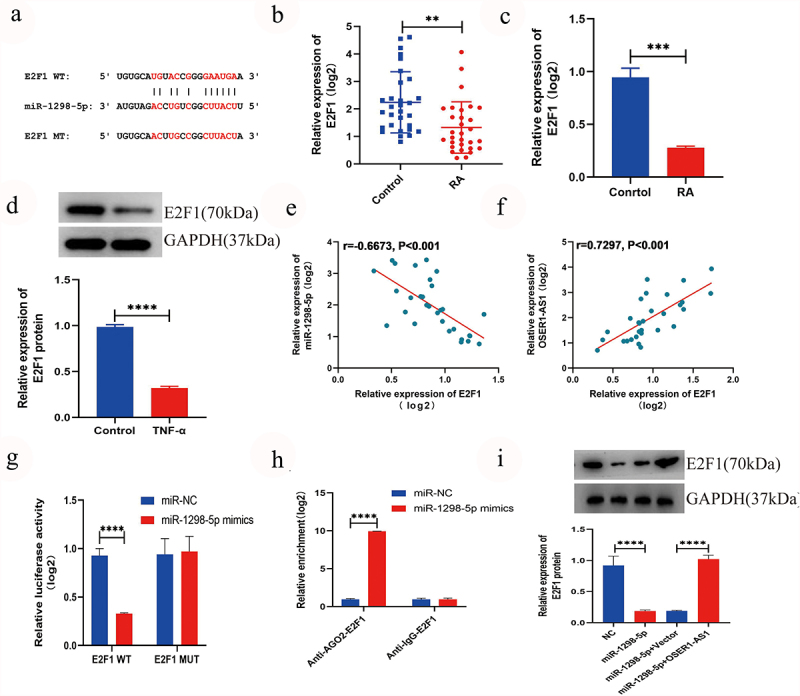
miR-1298-5p targeted E2F1 and had a negative correction with E2F1. (a) Bingding sites between miR-1298-5p and E2F1 were predicted by TargetScan. (b) The expression level of E2F1 in RA synovial tissues (n = 30) and compared normal synovial tissues (n = 30) was measured by qRT-PCR assay. (c) E2F1 expression was signifcantly lower in TNF-α-induced RA-FLSs. (d) The expression level of E2F1 protein in RAFLSs compared with normal synovial cells was detected by RT-qPCR. (e-f) Pearson correlation analysis was used to analyze the expression association between E2F1 and miR-1298-5p or OSER1-AS1 in RA synovial tissues. (g) Dual-luciferase reporter assay was used to detect the effect of miR-1298-5p-mimics on luciferase activity of E2F1-wt or E2F1-mut reporters in RAFLSs. (h) RIP assay was used to confirm the binding between miR-1298-5p and E2F1 in RAFLSs. (i) Western blot assay was applied to examine the protein level of E2F1 in RAFLSs transfected with NC, miR-1298-5p mimics, miR-1298-5p+pcDNA and miR-1298-5p mimics+pcDNA-OSER1-AS1. All experiments were conducted in triplicate. **P < 0.01, ***p < 0.001, ****p < 0.0001.

The result of TargetScan suggested that E2F1 may be a direct objective of miR-1298-5p [17] (Figure 4a). Furthermore, the outcome of RT-qPCR displayed that the expression level of E2F1 in RA synovial tissues samples (Figure 4b) and TNF-α-induced RA-FLSs (Figure 4c) was signifcantly lower. Western blot confirmed that the expression extent of E2F1 was lower expressed in RA-FLSs (Figure 4d). Association analysis displayed a negative relationship between the expression extents of miR-1298-5p and E2F1 in synovial tissue samples of RA patients (Figure 4e, r = −0.6673, p < 0.001), while a positive relationship between the expression extent of OSER1-AS1 and E2F1 was found (Figure 4f, r = 0.7297, p < 0.001). In addition, the dual-luciferase reporter assays were made to investigate the binding between E2F1 and miR-1298-5p and the outcomes displayed that the luciferase activity significantly declined during the co-transfection with E2F1-wt and miR-1298-5p mimics, whereas no significantly alternation was observed in luciferase activity in E2F1-mut and miR-1298-5p mimics group (Figure 4g). Moreover, the binding in RAFLSs (Figure 4h) was confirmed with RIP assay. The protein extent of E2F1 in RAFLSs transfection, miR-1298-5p+pcDNA and miR-1298-5p mimics+pcDNA-OSER1-AS1 was examined with Western blot assay, so as to dig the effect of miR-1298-5p mimics and pcDNA-OSER1-AS1 on E2F1. And the results confirmed the negative association between miR-1298-5p and E2F1 and OSER1-AS1 can reverse the effect (Figure 4i).

### OSER1-AS1 overexpression reverse the role of miR-1298-5p in E2F1 expression, the proliferation, release of inflammatory factor and cell apoptosis of TNF-α-caused RA-FLSs

**Figure 5.**
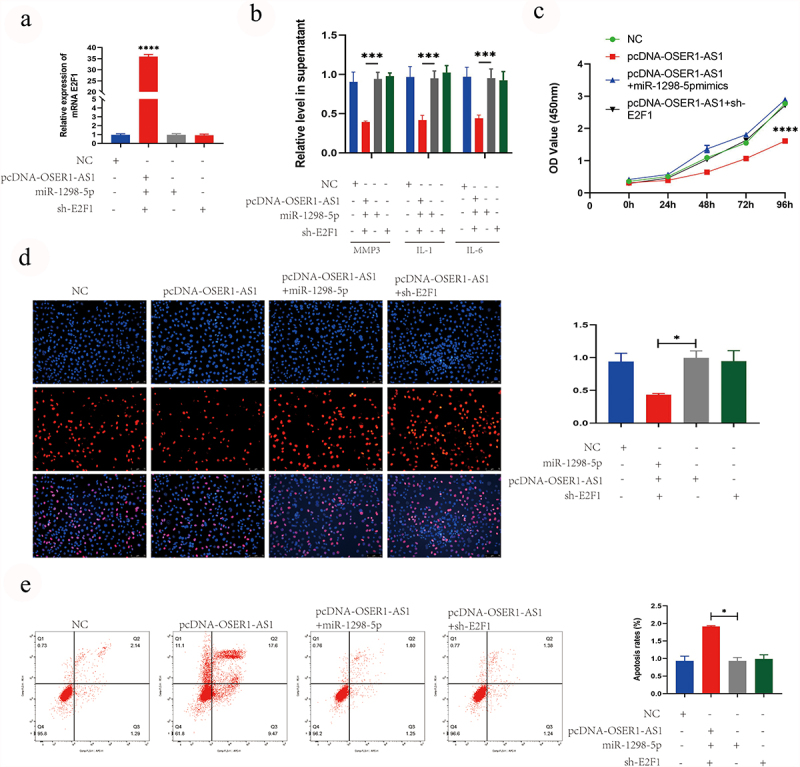
OSER1-AS1 overexpression reverse the effect of miR-1298-5p on E2F1 expression, the proliferation, release of inflammatory factor and cell apoptosis of TNF-α-induced RA-FLSs. (a) qRT-PCR analysis was performed to assess the overexpression efficiency of E2F1 after transfected with NC, pcDNA-OSER1-AS1, pcDNA-OSER1-AS1+ miR-1298-5p mimics and pcDNA-OSER1-AS1+ sh-E2F1. (b) ELISA kits were applied to detected the effect of OSER1-AS1, miR-1298-5p and E2F1 on production of interleukin-1 (IL-1), interleukin-6 (IL-6), matrix metalloproteinases-3 (MMP-3). (c-d) The inhibition effect of OSER1-AS1 on cell proliferation of the TNF-α-induced RA-FLSs was partially reversed by miR-1298-5p mimics and sh-E2F1 detected by CCK8 assay and EdU staining. (d) The promotion effect of OSER1-AS1 on cell apoptosis of the TNF-α-induced RA-FLSs was partially reversed by miR-1298-5p mimics and sh-E2F1 detected by Flow cytometry apoptosis assay. All experiments were conducted in triplicate. ***p < 0.001, ****p < 0.0001.

Finally, the effect of the OSER1-As1/miR-1298-5p/E2F1 axis on the proliferation and apoptosis was analyzed. The outcomes of RT-qPCR analysis illustrated that the levels of E2F1 was significantly higher after transfected with pcDNA-OSER1-AS1, compared with NC, pcDNA-OSER1-AS1+ miR-1298-5p mimics and pcDNA-OSER1-AS1+ sh-E2F1 (Figure 5a). The results of ELISA assay indicated that the inhibition effect on the release of RA-induced inflammatory production of IL-1, IL-6 and MMP3 by overexpressing OSER1-AS1 was reversed by miR-1298-5p mimics and sh-E2F1 (Figure 5b). The inhibiting role of OSER1-AS1 in cell proliferation of the TNF-α-induced RA-FLS was partly reversed by miR-1298-5p mimics and sh-E2F1 detected by CCK8 assay and EdU staining (Figure 5c-d). The promoting role of OSER1-AS1 in cell apoptosis of the TNF-α-induced RA-FLS was partly reversed by miR-1298-5p mimics and sh-E2F1 detected by Flow cytometry apoptosis assay (Figure 5d).

## Discusion

The present research indicated that OSER1-AS1 was greatly downregulated in RA serum and tissues and OSER1-AS1 can act as a potential promising diagnostic biomarker. The result of ROC analysis showed that the level of OSER1-AS1 in blood and synovial tissues can tell apart RA patients from healthy controls, of which sensitivity and specificity was superior than rheumatoid factor (RF) and anti-cyclic citrullinated peptide (anti-CCP) [22]. Overexpression of OSER1-AS1 inhibited the propagation and inflammation of RA-FLS, and drove RA-FLS apoptosis. Mechanistically, we found that overexpression of OSER1-AS1 suppressed the inflammation and apoptosis of RA-FLS via regulating miR-1298-5p/E2F1 axis.

Accumulating evidence indicated that lots of lncRNAs were differentially expressed in RNA and lncRNAs participated in the onset and development of RA. For example, Zhang and coworkers observed the high exprssion oflncRNA SNHG14 in RA and SNHG14 knockdown inhibited pTHP-1 cell propagation and proinflammatory cytokines generation through miR-17-5p/MINK1-JNK pathway [23]. Liu and colleagues showed that NEAT1 from serum-derived exosomes boosted the progression of RA via miR-144-3p/ROCK2 axis [24]. Liu *et al*. demonstrated the up-regulation of lncRNA SNHG1 in RA-FLSs by comparing with FLSs from trauma arthritis and osteoarthritis patients and SNHG1 accelerated the propagation, migration and aggression of RA-FLSs by interaction with PTBP1 [25]. Robin Caire et al reported that synovial membrane stiffening was enhanced by YAP/TAZ transcriptional activity [26]. These studies indicated lncRNA exerted a critical effect on the pathogenesis of RA. The current research identified the great down-regulation of OSER1-AS1 in RA serum and tissues. Furthermore, we found that overexpression of OSER1-AS1 inhibited the propagation and proinflammatory cytokines production and boosted the apoptosis of TNF-α-induced RA-FLS. Collectively, these findings indicated that low expression of OSER1-AS1 served as a significant contributor in the progression of RA.

Increasing studies showed that lncRNAs exert their functions through sponging miRNAs in RA. For instance, NEAT1 promoted RA-FLSs cell proliferation and inflammatory cytokine production through sponging miR-204-5p [27]. ZFAS1 silencing alleviated the progression of RA through inhibiting miR-296-5p [28]. Similarly, many studies also revealed that OSER1-AS1 participated in regulating the progression of cancers through binding to miRNAs. Shi *et al*. found that OSER1-AS1 boosted gefitinib resistance of lung adenocarcinoma via miR-612/FOXM1 pathway [29]. Liu and colleagues showed that OSER1‑AS1 acted as a sponge of miR‑433‑3p to boost cell propagation, move, and aggression in non‑small cell lung cancer [6]. Also, OSER1-AS1 served as a ceRNA for miR-372-3p to increase the expression of Rab23, thereby promoting tumorigenesis in hepatocellular carcinoma [7]. The present research illustrated that miR-1298-5p existed potential binding OSER1‑AS1. Moreover, our outcomes dislayed that miR-1298-5p was upregulated in RA tissues by comparing with control group and the expression of OSER1‑AS1 was negatively related to miR-1298-5p. These outcomes displayed that miR-1298-5p was the downstream objective for OSER1‑AS1 in RA. Subsequently, miR-1298-5p could partialy reversed the role of OSER1‑AS1 overexpression in RA-FLSs.

In general, miRNAs exert their biological functions through binding to targeted mRNAs. Our results revealed that E2F1 might be a direct objective of miR-1298-5p through bioinformatics analysis. Also, E2FI was greatly downregulated in RA tissues and TNF-a-treated FLSs. A negative relationship between the expression of E2F1 and miR-1298-5p was observed. These outcomes showed that E2F1 was the direct downstream objective for miR-1298-5p in RA. Previous studies showed that E2F1 was significantly downregulated in RA, and overexpression of E2F1 suppressed the proliferation and invasion of FLS and pro-inflammatory cytokines production via p53 signaling pathway in RA [27]. In our study, we found that downregulation of E2F1 was partly induced by OSER1-AS1 inhibition through upregulating miR-1298-5p, which suggested that OSER1-AS1 exerted its regulatory function via miR-1298-5p/E2F1 axis in RA.

## Conclusions

Our results uncovered that OSER1-AS1 was significantly downregulated in RA. Overexpression of OSER1-AS1 inhibited FLSs proliferation and pro-inflammatory cytokines production, and promoted apoptosis in RA. OSER1-AS1 acted as the sponge of miR-1298-5p and E2F1 was the downstream targeted mRNA of miR-1298-5p. Further result revealed that OSER1-AS1 regulated the biological processes in RA-FLSs via miR-1298-5p/E2F1 axis. The current findings identified that OSER1-AS1/ miR-1298-5p/E2F1 axis exerted a significant effect on pathogenesis of RA, which may offer novel diagnostic and therapeutic targets for RA.

## Supplementary Material

Supplemental Material

## Data Availability

All data generated or analyzed during this study are available in this article or supplementary material.

## Highlights

- OSER1-AS1 was lowly expressed in serum and synovial tissues of RA patients and compared healthy controls.
- OSER1-AS1 inhibited the proliferation, release of inflammatory factor and boosted the apoptosis of TNF-α-induced RA-FLSs.
- OSER1-AS1 regulated the proliferation, release of inflammatory factor and cell apoptosis of TNF-α-induced RA-FLSs via modulating miR-1298-5p/E2F1 axis.
- OSER1-AS1 may provide novel diagnostic and therapeutic targets for RA.
