# Supplementary figures and images for: LncRNA OSER1-AS1 regulates the inflammation and apoptosis of rheumatoid arthritis fibroblast like synoviocytes via regulating miR-1298-5p/E2F1 axis
Source: Bioengineered. 2022 Feb 14;13(3):4951–63. doi: 10.1080/21655979.2022.2037854 (PMC8974142; doi:10.1080/21655979.2022.2037854)

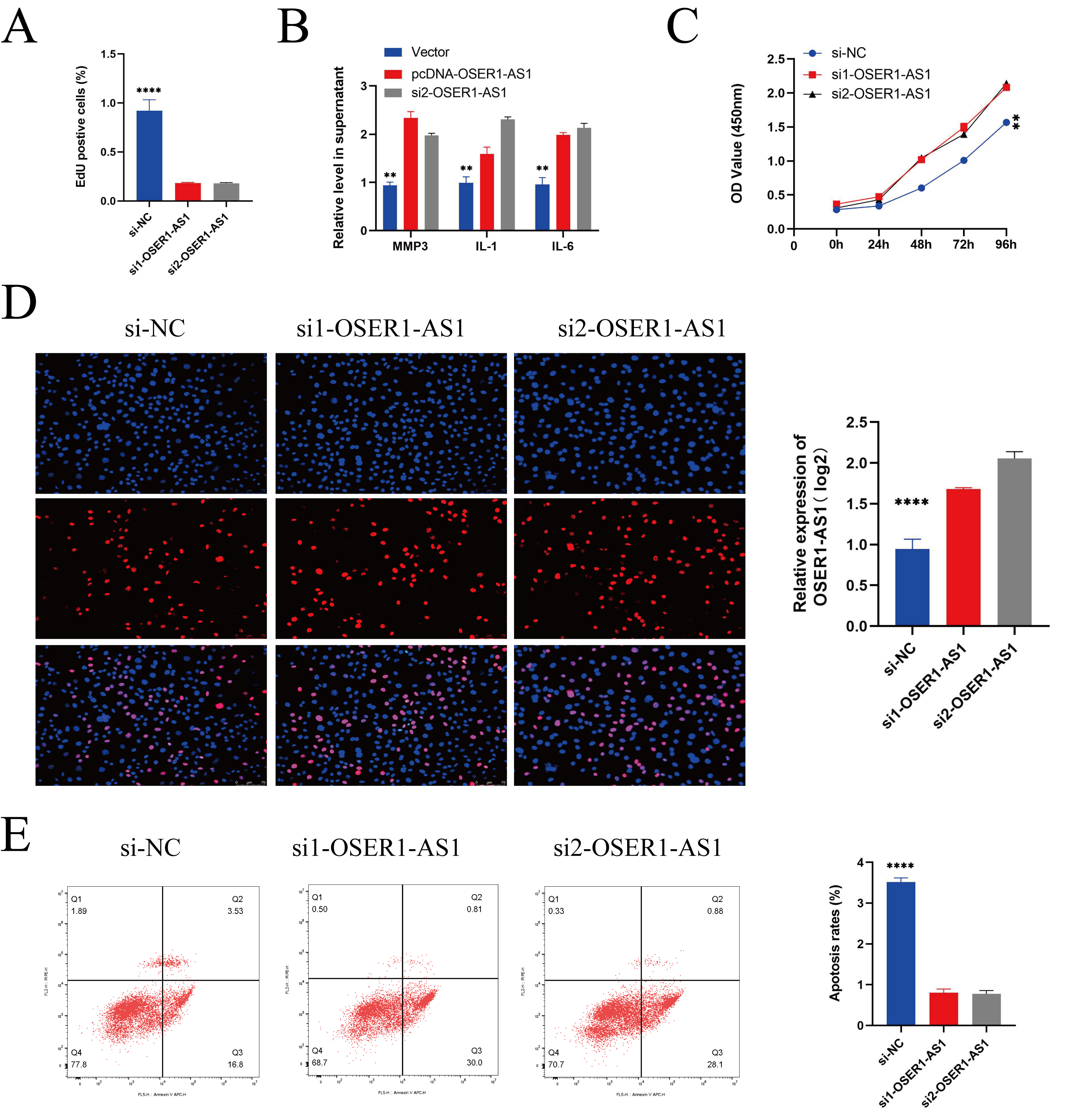

Supplement: Supplemental Material [file KBIE_A_2037854_SM1861.tif]
